# Helmholtz at 200

**DOI:** 10.1177/20416695211022374

**Published:** 2021-07-02

**Authors:** Nicholas J. Wade

**Affiliations:** Psychology, University of Dundee, Dundee, UK

**Keywords:** Helmholtz, anaglyphs, physiological optics, vision, stereopsis, rivalry, lustre, art

## Abstract

Hermann von Helmholtz was born 200 years ago, but his influence on vision research is enduring. His legacy in vision is celebrated visually with anaglyphs that combine portraits of him with illustrations from his publications. Emphasis is directed principally to his *Treatise on Physiological Optics*. Among the optical instruments Helmholtz invented were the ophthalmoscope, ophthalmometer, and telestereoscope. Mention is made of his investigations into accommodation, colour vision, eye movements, stereoscopic vision, binocular rivalry, and lustre. Helmholtz also presented his analyses of vision and art in several of his *Popular Lectures.*

Hermann Ludwig Ferdinand von Helmholtz was born on August 31st, 200 years ago. So much has been written about him both in terms of his life and his science that Kremer (1994) referred to it as the “Helmholtz industry.” It is sure to grow in this year marking the bicentenary of his birth. The many biographies of Helmholtz (1821–1894) span the period between those of M'Kendrick (1899) and Cahan (2018). A brief biography is given in *Perception*, as is an account of the passage of the *Handbuch* through its three editions to its translation (Wade, 1994, 2004). In *Perception* and *i-Perception*, Helmholtz has been cited in 613 articles, 15 of which include his name in their titles. Most of the citations are to his *Handbuch der physiologischen Optik* which was published in 1867. The centenary of his birth was marked by the translation of his *Handbuch* into English as *Helmholtz’s Treatise on Physiological Optics* (Helmholtz, 1924a, 1924b, 1925). A detail from the frontispiece portrait of Helmholtz from the first volume of the translation is shown as part of an anaglyph in Figure 1, together with the title page.

**Figure 1.**
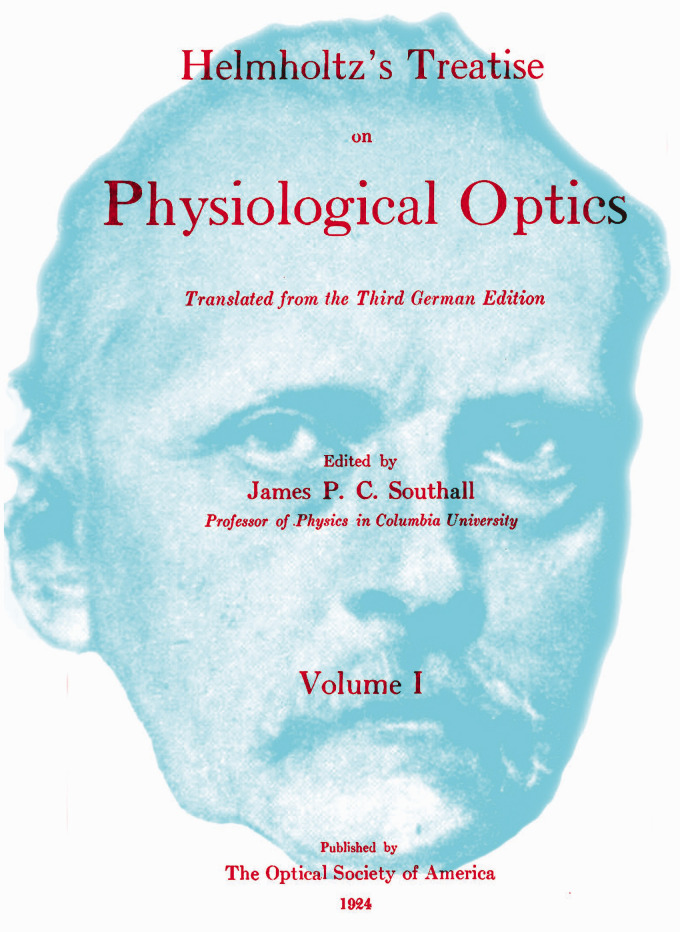
*Helmholtz’s Treatise* by Nicholas Wade. Title page of Southall’s translation of the *Handbuch* into English. The portrait of Helmholtz in 1876 is derived from a frontispiece lithograph in the *Treatise*.

The first stereoscopic colour separations were made by a contemporary of Helmholtz (Rollmann, 1853), although they were not called anaglyphs until later in the century (see Wade, 2021a). They were mentioned in the *Handbuch*:

He [Rollmann] draws two projections on the same black card, one with red lines, the other with blue. Then he takes a red glass in front of one eye and a blue glass in front of the other and only sees the red lines with that eye, with this only the blue ones, which can then be combined to form a relief. (Helmholtz, 1867, p. 685)

That is, the red lines are seen as black through the blue glass as are the blue lines through the red glass. It seems to have gone unnoticed that this text was incorrectly translated by Southall who wrote “ . . . he can see only the red lines through the red glass and the blue lines through the blue glass” (Helmholtz, 1925, p. 356). Anaglyphs are displays in which the left and right eye images are printed in different colours, such as red and cyan, and they are viewed through filters of the same colours. They have typically been used to present slightly different images to each eye so that they are seen in stereoscopic depth, but they can also be enlisted to demonstrate binocular rivalry and a range of other dichoptic interactions (see Wade, 2021b). Anaglyphs do not separate the patterns to each eye as fully and equally as optical stereoscopes, but they do have some advantages. The superimposition of red and cyan images creates a third image that can have an allure of its own (see Wade, 2021b). The ease with which the components can be viewed alone (by closing or covering one eye while using the filters) displays the monocular elements and (for stereoscopic or rivalry patterns) acts as an efficient technique for presenting two patterns in a single image.

## Helmholtz’s *Treatise*

There were three editions of the *Handbuch* in German (Helmholtz, 1867, 1896, 1909, 1910, 1911). The first two were single volumes, but the third was in three volumes. Willibald Nagel, together with Allvar Gullstrand and Johannes von Kries, based the third edition of the *Handbuch* on Helmholtz’s text from the first edition of 1867, rather than on the revised second edition of 1896. When considering the difficulties of preparing a third edition, Nagel wrote:

Under the circumstances there was nothing else to be done but to preserve the text of the original work intact, and at the expense of a certain unity and uniformity in the work as a whole to limit the revision to supplementary chapters. (Helmholtz, 1924a, p. xi)

**Figure 2.**
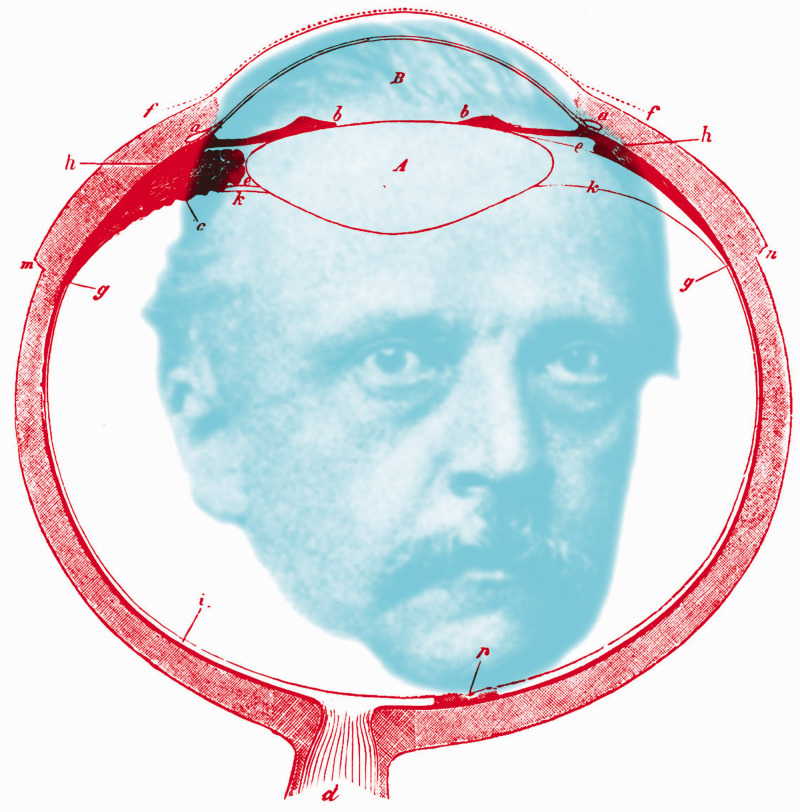
*Helmholtz’s eye* by Nicholas Wade. Helmholtz in 1867, the year in which the three parts of his *Handbuch* were published in a single volume. The portrait is after a photograph in Hall (1912) and the diagram of the eye is after Figure 2 in Helmholtz (1924a).

The editors added footnotes, notes, and additional references to many of the sections, and each wrote extensive appendices based largely upon their own experimental researches. Thus, Helmholtz’s text that is translated into the English *Treatise* is from the first edition, even though it was derived from the third German edition. Volume 1 of the *Treatise* describes the anatomy and optics of the eye, with consideration of image formation and optical aberrations. Volume 2 treats the sensations of vision, dealing principally with colour and contrast phenomena. Volume 3 is entitled the theory of visual perception, and it addresses eye movements, visual direction, and binocular vision. Essentially, they were concerned with the physics, physiology, and psychology of vision, respectively. Helmholtz is shown in Figure 2 surrounded by his diagram of the human eye from the *Treatise*.

**Figure 3.**
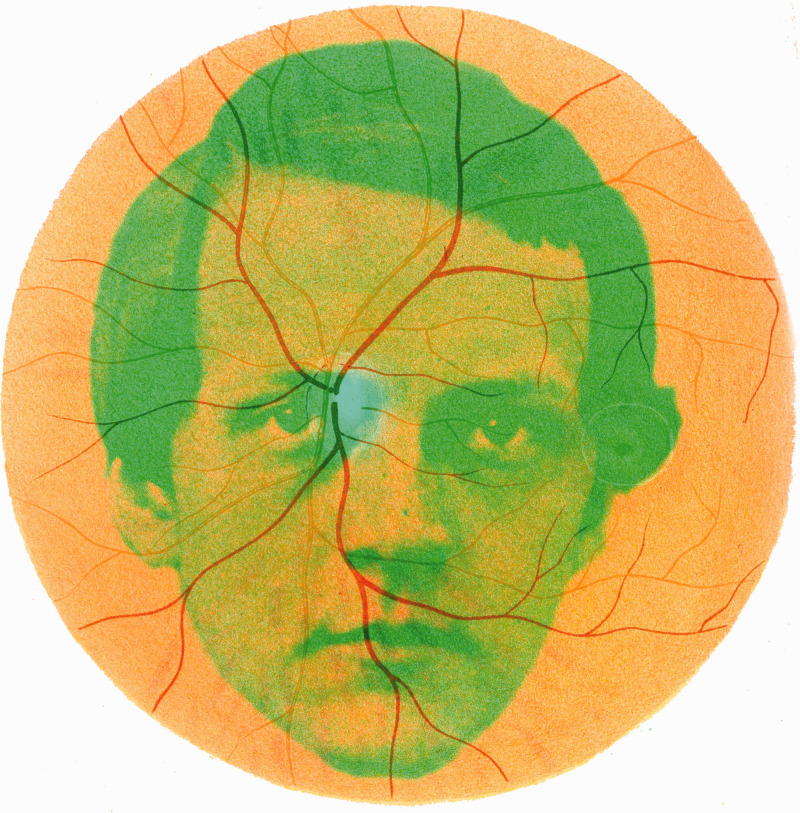
*Helmholtz’s retina* by Nicholas Wade. A daguerreotype of Helmholtz from 1848 and a coloured illustration of the retina. The portrait is derived from Koenigsberger (1902) and the retina as seen with an ophthalmoscope is Figure 1, Plate 2 in Helmholtz (1896).

The illustrations in the *Handbuch* did change over the three editions. In the first edition, there were 213 woodcuts in the text to which were appended 11 Plates; in the second, there were 254 text illustrations and 8 Plates. The text figures were numbered consecutively throughout the volumes. The third edition broke away from this pattern and numbered the pages and text figures separately for each volume: There were 146 text illustrations in Volume 1, 80 in Volume 2 with three Plates, and 81 in Volume 3 with six Plates. The final six Plates were the same in all editions, and five of them illustrated stimuli for binocular vision. Those not reproduced in the third edition concerned the gross and microscopic structure of the eye and retina, diagrams of optical instruments and of some entoptic phenomena. Although *Treatise* was based on Helmholtz’s text from the first edition, the nine Plates reprinted were from the third edition. Plates 1 and 2 of the second edition were in colour and included a representation of the retina as seen with an ophthalmoscope. A young Helmholtz is shown in Figure 3 together with the colour illustration of the retina as seen with an ophthalmoscope.

## Monocular Vision

**Figure 4.**
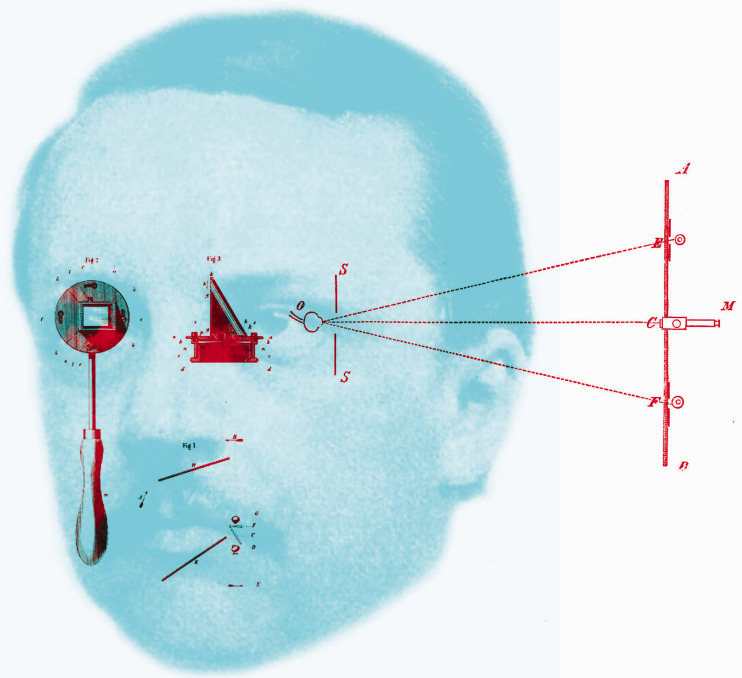
*Helmholtz’s ophthalmoscope and ophthalmometer* by Nicholas Wade. The portrait is after a photograph taken in 1857. Diagram of the ophthalmoscope and its operation (on the left) is from Helmholtz (1851) and that of the ophthalmometer (on the right) is from Helmholtz (1855).

Helmholtz (1851, 1916) added to the instrumental revolution in studies of vision with his invention of the ophthalmoscope. It met with instant acclaim and revealed a new world to ophthalmologists, assisting greatly in the diagnosis and treatment of eye ailments. Many modifications and improvements of Helmholtz’s model were made during his lifetime (Keeler, 2002). The ophthalmoscope not only transformed the clinical examination of the eye, but it also led to the creation of a new journal in 1854, the *Archiv für Ophthalmologie* edited by Albrecht von Graefe. It was in this journal that Helmholtz announced his ophthalmometer and described its application to measuring the changes in lens curvature that accompany accommodation (Helmholtz, 1855). The article was published in the second issue of the first volume of the journal; although the first issue was published in March 1854, the second was published in the following March, and the pages were numbered separately. Helmholtz is shown in Figure 4 with his diagrams of the ophthalmoscope and the ophthalmometer.

The ophthalmoscope and ophthalmometer sparked Helmholtz’s interests in physiological optics, and it ushered in almost two decades of research on the senses. They were also part of an experimental transformation that had engulfed the study of vision during the previous two decades, and Helmholtz was able to capitalise on it. Experiments could be performed on vision, and its study was transferred from the natural environment to the laboratory where the methods of physics could be applied (see Wade, 1998, 2016; Wade & Heller, 1997). The ophthalmometer enabled Helmholtz to tackle the perplexing problem of accommodation. To measure the curvatures of the optical surfaces in the living eye, he confirmed the speculations that the crystalline lens changes curvature during accommodation, and he proposed a mechanism by which this is achieved:

On contraction, the ciliary muscle could pull the posterior end of the zonule forwards nearer the lens and reduce the tension of the zonule. . . .If the pull of the zonule is relaxed in accommodating for near vision, the equatorial diameter of the lens will diminish, and the lens will get thicker in the middle, both surfaces becoming more curved. (1924a, p. 151).

**Figure 5.**
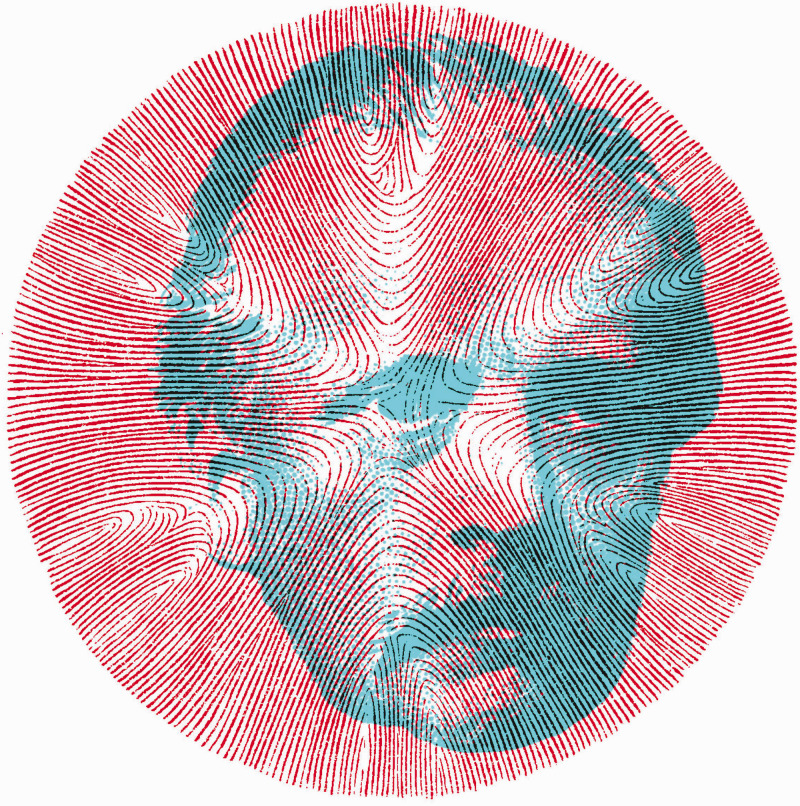
*Helmholtz’s crystalline lens* by Nicholas Wade. A portrait of Helmholtz in 1867 combined with a diagram showing the “characteristic star-shaped figures . . . from the outer layers of the lens” (Helmholtz, 1924a, p. 33). The portrait is derived from an engraving in Koenigsberger (1902); the diagram of the crystalline lens is after Figure 20 in Helmholtz (1924a).

Figure 5 represents an older Helmholtz within the structure of the lens as illustrated in the *Treatise*.

**Figure 6.**
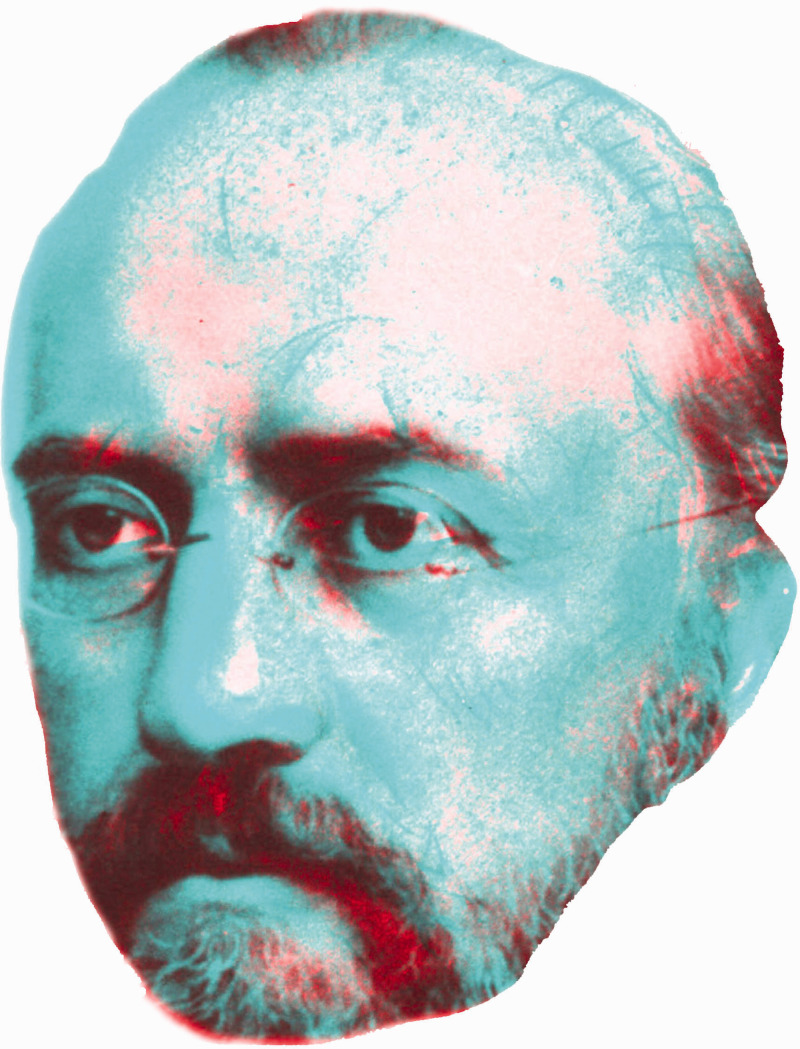
*Rivalry between Helmholtz and Hering* by Nicholas Wade. The portrait of Helmholtz is after a sketch made in the year of his death (from Koenigsberger, 1903) and the portrait of Hering is derived from an illustration in Pagel (1901).

The material on accommodation was incorporated and enlarged in the first volume of the *Treatise*, but the analyses of colour and contrast were presented in the second volume. Helmholtz differentiated between additive and subtractive colour mixing and became an ardent proponent of Young’s (1802) trichromatic theory following publication of more detailed support for it by Maxwell (1855) using his colour disc. It was also an area in which he engaged in a bitter rivalry with Ewald Hering (1861, 1862) who advanced an opponent process theory (see Howard, 1999; Turner, 1994). The disputes between Helmholtz and Hering (see Figure 6) were not limited to colour vision but were also expressed in their interpretations of binocular colour combination and stereoscopic vision. Studies of binocular colour combination have a long history, and disparities between those who reported rivalry and those who saw colour mixtures were voiced long before the stereoscope was invented (see Wade & Ngo, 2013). However, binocular colour combination could be investigated more easily with the aid of a stereoscope, and Wheatstone (1838) described the fluctuations in the visibility of the different colours presented to each eye. The changes in visual awareness corresponded to his inferential theory of vision in which binocularity was considered to be psychological rather than physiological. Helmholtz followed Wheatstone theoretically, and he also observed colour rivalry rather than colour mixture. Hering (1861), on the other hand, argued for a physiological interpretation of the colour combinations he observed, and much of the dispute surrounded the visibility of yellow from dichoptic combinations of red and green.

## Binocular Vision

The long history of investigations of binocular vision was revolutionised by the invention of the stereoscope and the experience of depth with stimulation of non-corresponding points. Helmholtz addressed the topic in Volume 3 of the *Treatise*. He commenced by examining visual direction: Monocular vision could signal direction alone, but location required distance which could be supplied by binocular vision. In this context, he introduced the term *cyclopean eye* (see Wade, 2021a). The concept is based on the mythological cyclops who forged thunderbolts for Zeus and was a one-eyed giant in the Homeric *Odyssey*. The direction in which objects were seen with two eyes was as if the origin was a point between them.

Prior to the invention of the stereoscope, vision with two eyes was considered in terms of singleness rather than depth. Two contrasting approaches were proposed for single vision—suppression or fusion (see Wade & Ono, 2012). Helmholtz’s mentor, Johannes Müller (1826, 1838), considered that fusion was restricted to stimuli lying on a circle with its circumference based on the point of fixation with both eyes and the rotation centres of each of them; it became called the Vieth-Müller circle because Vieth (1818) gave a similar geometrical account of binocular single vision. All points on the circle stimulated corresponding or identical retinal points yielding single vision; all other patterns of binocular stimulation were considered to lead to double vision. This was questioned by the stereoscopic phenomena described by Wheatstone (1838): “objects whose pictures do not fall on corresponding points of the two retinæ may still appear single” (p. 384). The implications of this to received theory were summarised by Volkmann:

As is well known, Charles Wheatstone the famous inventor of the stereoscope had drawn the conclusion from his experiments that the doctrine of the identity of the retina, as established by physiological optics, is not tenable. If Wheatstone was right on this point, the doctrine of vision was threatened to be overturned completely. For one only needs to remember Johannes Müller’s excellent investigations into the identical retinal points and their positions in order to see that with the abandonment of the doctrine of identity, the doctrine of double images, of the horopter and the apparent distance between two images in the fields of vision collapse, precisely those doctrines which are based on exact experiments, namely experiments that can be controlled by measurements and numbers. It is therefore very natural for the physiologists to counter Wheatstone’s conclusions and try to reconcile the phenomena he observed with the theory of identical retinal sites. (1859, pp. 1--2; 2020, p. 223)

There was no doubting the phenomena of stereoscopic depth perception but interpretations of them differed. It could be argued that the stereoscope heralded a revolution in vision, and the instrument was embraced by Helmholtz. He initiated research on binocular vision in the 1850s, although most of his experiments were undertaken in the early 1860s when in Heidelberg. The stereoscope was very important to Helmholtz, both for the experimental world it exposed and also for his inferential theory of vision. In prosecuting his experimental enquiries, Helmholtz (1857) developed the reflecting stereoscope so that disparities could be enhanced. This was achieved by extending the separations between the mirrors, and he called the instrument a telestereoscope; his diagram of it is shown together with his portrait in Figure 7. In the *Treatise*, he described modifications of Brewster’s refracting stereoscope so that the degree of convergence could be adjusted, and he improved on the design of the telestereoscope (Helmholtz, 1925, pp. 350–352).

**Figure:**
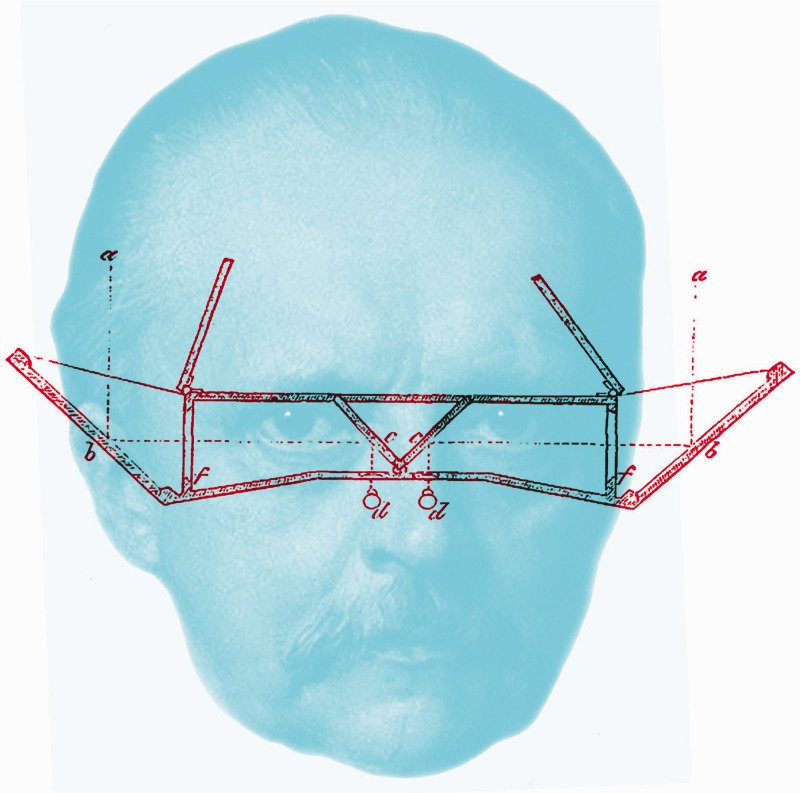
Figure 7. *Helmholtz’s telestereoscope* by Nicholas Wade. The portrait is modified from a painting dated 1881 and the diagram of the telestereoscope is from Helmholtz (1857).

Eye movements were implicated in stereoscopic vision by Brücke (1841), a close associate of Helmholtz, to reconcile the binocular phenomena with Müller’s theory of identical retinal points. If the eyes changed convergence rapidly while viewing solid objects, then this could be the basis for perceived depth rather than the combination of slightly disparate retinal points in the two eyes. While Helmholtz adopted eye movement interpretations of many visual effects, this did not apply to depth seen with two eyes. He confirmed the occurrence of stereoscopic vision without eye movements. This was demonstrated in two ways. First, brief electrical sparks which illuminated stereoscopic pairs generated afterimages and depth was visible with them. Second, presenting paired stimuli briefly with a tachistoscope also resulted in stereoscopic depth. Helmholtz wrote:

Both these experiments and those with electric sparks show that ocular movements are not necessary for perception of depth; because after-images move with every movement of the eye, and it is simply impossible to make disparate images correspond to each other by any such movement. (1925, p. 456)

The stereoscope was used by Helmholtz to examine binocular colour and contour rivalry as well as the perception of depth. Hering (1861) considered that binocular rivalry was physiological, whereas Wheatstone and Helmholtz maintained that it was psychological; this would now be described as a difference between peripheral and central interpretations. In his *Treatise*, Helmholtz discussed rivalry in some detail and also emphasised that changing, complex mixtures of the two stimuli tend to be visible most of the time with only occasional periods in which the stimulus in one eye alone dominates:

. . . in the various parts of the field, one image will prevail more than the other, whereas in other parts the other image will predominate. Sometimes there will be alternations, so that, where for a while only parts of one image were visible, presently parts of the other image will emerge and suppress portions of the first image. This fluctuation, in which parts of the two images mutually supplant each other, either side by side, or one after the other, is what is usually meant by *the rivalry between the visual globes*. (Helmholtz, 1925, p. 494)

**Figure 8.**
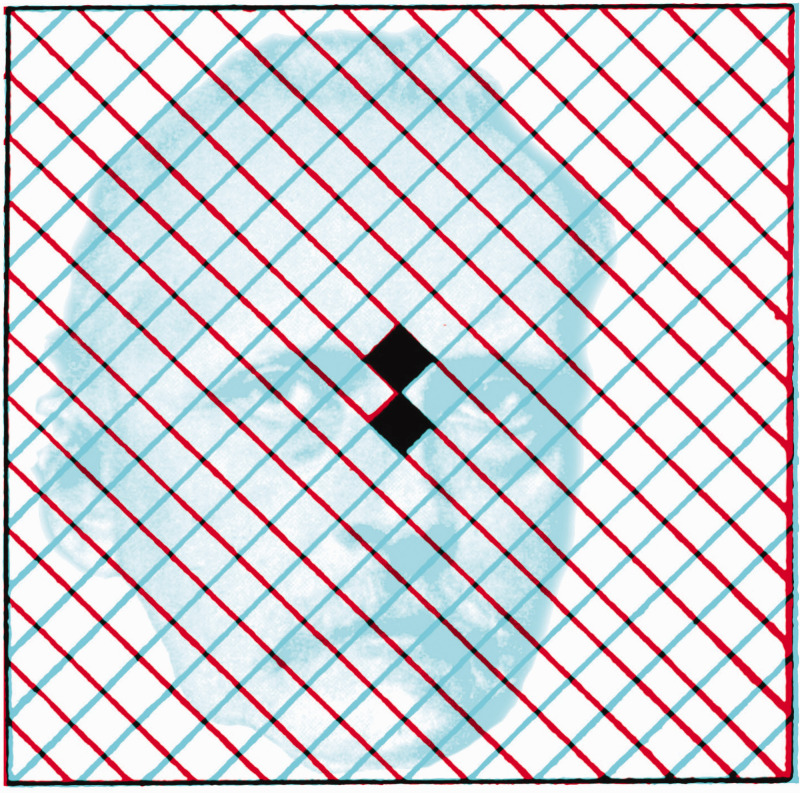
*Helmholtz in rivalry* by Nicholas Wade. Helmholtz is shown in combination with the crossed gratings that he illustrated in Plate 6 of his *Treatise* (Helmholtz, 1925).

Helmholtz placed great importance on eye movements in binocular rivalry and made a modification to Panum’s (1858) orthogonal gratings configurations: He placed two small squares at the centre of both gratings to facilitate common fixation by each eye (Figure 8) because otherwise “it is difficult to concentrate the attention on one of the systems of lines” (Helmholtz, 1925, p. 498). Thus, Helmholtz also assigned rivalry to attention: “The extraordinary influence exercised by contours in the rivalry between the two visual globes is also essentially a matter of psychological habit, in my opinion” (Helmholtz, 1925, p. 501).

Prior to Panum, rivalry patterns presented in a stereoscope were complex like letters (see Wade, 2019, 2021a). He introduced the crossed gratings because “The rivalry of contours is at its strongest if the different lines in the two images are as equal as possible with regard to thickness and light intensity” (Panum, 1858, p. 38). The crossed diagonal figure was used by Helmholtz to support his theory that rivalry is a psychological rather than a physiological process because he could control which stimulus was visible:

These experiments show that man possesses the faculty of perceiving the images in each eye separately, without being disturbed by those in the other eye, provided it is possible for him . . . to concentrate his whole attention on the objects in this one field. This is an important fact, because it signifies, that *the content of each separate field comes to consciousness without being fused with that of the other field by means of organic mechanisms;* and that, therefore, *the fusion of the two fields in one common image, when it does occur, is a psychic act*. (Helmholtz, 1925, p. 499)

**Figure 9.**
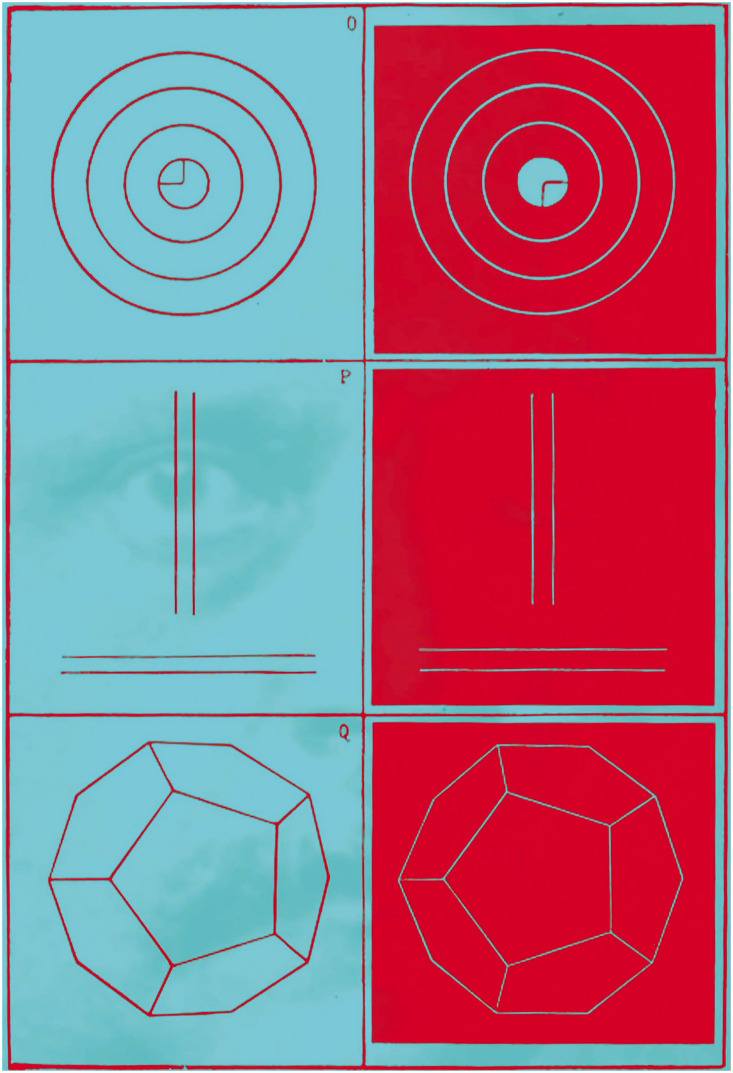
*Helmholtz and stereoscopic lustre* by Nicholas Wade. The portrait is derived from a frontispiece lithograph in Helmholtz (1925) as are the diagrams that induce stereoscopic lustre (Plate 4).

In 1839, the year following the announcement of the stereoscope by Wheatstone, photography was introduced to the public. Soon after, paired photographs from slightly different positions were taken that could be viewed in a stereoscope to yield apparent depth. It was also possible to present a positive picture to one eye and a negative to the other. However, the initial studies of binocular lustre by Dove (1851) used drawings of geometrical figures either as black on white or white on black. Helmholtz examined this aspect of binocular vision which he called “stereoscopic lustre” and stimuli inducing it were accorded the status of a Plate in his *Treatise* (Figure 9).

## Perception

**Figure 10.**
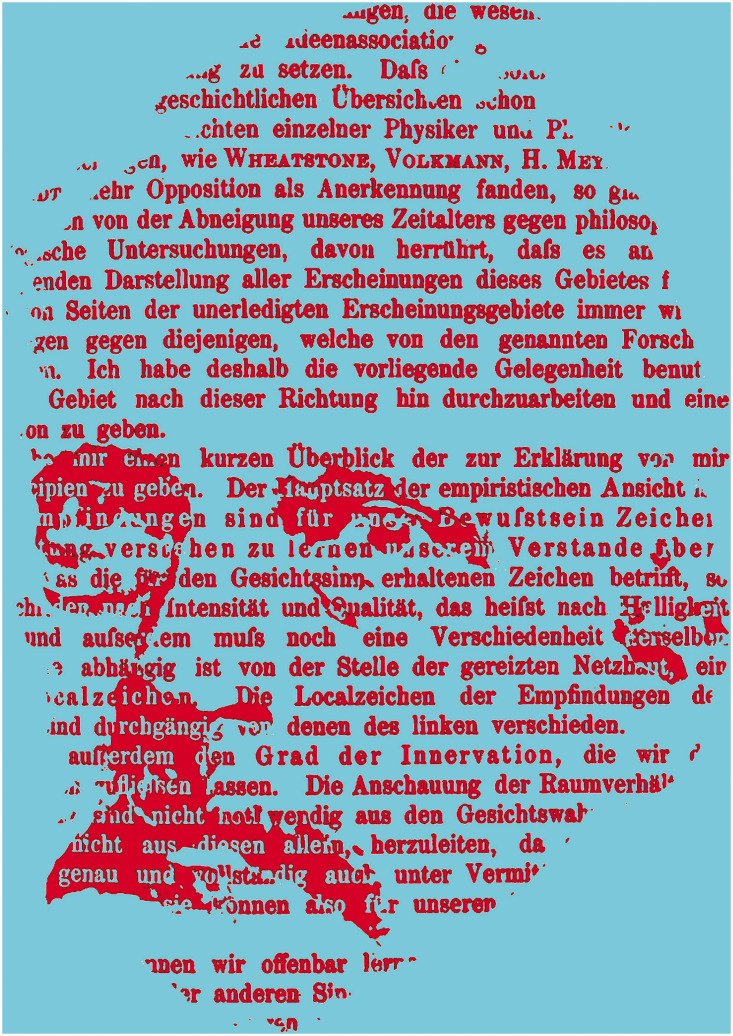
*Tokens of binocular sensation* by Nicholas Wade. A portrait of Helmholtz, after an engraving in Koenigsberger (1902), is embedded in text from the *Handbuch* (1896, p. 947). A translation of the text can be found on pp. 532–533 of Helmholtz (1925).

Despite describing the details of the eye as an optical instrument, colour and contrast sensations, and binocular vision, Helmholtz’s lasting influence in visual science has related to neither physics nor physiology of vision rather to his epistemology as it was developed in Volume 3 of the *Treatise* (see Hatfield, 1990; Turner, 1994). He acknowledged that little he wrote on the issue was novel, but he marshalled the arguments over a wider range of phenomena than others had done before. He summarised his position succinctly: “The fundamental thesis of the empirical theory is: *The sensations of the senses are tokens for our consciousness, it being left to our intelligence to learn how to comprehend their meaning*” (Helmholtz, 1925, p. 533). The German text for this carries Helmholtz’s portrait in Figure 10 which draws attention to the intelligence of perception, and the reader requires to exercise inference to extract Helmholtz’s portrait from the text.

In developing the theory that sensory signs (or tokens) rather than images are the basis for perception, Helmholtz was guided by Müller’s doctrine of specific nerve energies (see Cassedy, 2008; De Kock, 2016; Finger & Wade, 2002). Müller (1826, 1838) argued that the particular sensations experienced are dependent on the nerves excited, no matter how those nerves are stimulated. Therefore, vision does not reflect the nature of the stimulus but the stimulation of the optic nerve—it is indirect. For Helmholtz, these sensory signs were processed rapidly, unconsciously, and inferentially in what came to be called “unconscious inferences.” A similar concept was advanced by Berkeley (1709), and it was employed by Wheatstone (1838) to interpret stereoscopic phenomena. It was also applied by Oppel (1855) in his analysis of geometrical optical illusions (see Wade et al., 2017). Helmholtz’s many debts to Wheatstone were acknowledged in his *Handbuch*, as can be seen in the text of Figure 10. Not only did Wheatstone provide the instrument with which Helmholtz would challenge the nativism of Hering, he also supplied the theoretical framework, including the concept of unconscious mental processes, which was used to interpret stereoscopic observations. Wheatstone effectively reunited binocularity with space perception, and he employed Berkeleyian empiricism to cement the union. Space perception had remained the province of philosophers for centuries, but it was clear to both Wheatstone and Helmholtz that the methods of physics could be applied in an effective way to study space and depth perception; they were not, as Kant (1781) had contended, outside the realm of experimental enquiry.

Helmholtz was refuting the image-based theories that had long been dominant following Kepler’s (1604) analysis of ocular dioptrics. Kepler demonstrated that an inverted and reversed image of external objects was focussed on the back of the eye; he referred to it as a picture and set in train an approach to vision which involved processing this retinal picture. This created problems such as relating upright vision to an inverted image. As was the case with single vision from two retinal images, such problems were bypassed when considering sensory signs:

These two difficulties do not apply to the Empirical Theory, since it only supposes that the actual sensible “sign,” whether it be simple or complex, is recognised as the sign of that which it signifies. An uninstructed person is as sure as possible of the notions he derives from his eyesight, without ever knowing that he has two retinae, that there is an inverted picture on each, or that there is such a thing as an optic nerve to be excited, or a brain to receive the impression. He is not troubled by his retinal images being inverted and double. He knows what impression such and such an object in such and such a position makes on him through his eyesight, and governs himself accordingly. But the possibility of learning the signification of the local signs which belong to our sensations of sight, so as to be able to recognise the actual relations which they denote, depends, first, on our having movable parts of our own body within sight; so that, when we once know by means of touch what relation in space and what movement is, we can further learn what changes in the impressions on the eye correspond to the voluntary movements of a hand which we can see. In the second place, when we move our eyes while looking at a field of vision filled with objects at rest, the retina, as it moves, changes its relation to the almost unchanged position of the retinal picture. We thus learn what impression the same object makes upon different parts of the retina. (Helmholtz, 1873, p. 278)

In the German texts, Helmholtz referred to the sensory signals as “Zeichen” (see the text in Figure 10). It is clear from the aforementioned quotations that these became “tokens” for Southall in his *Treatise* and “signs” in the translation of Helmholtz’s *Popular Lectures* on “The recent progress of the theory of vision.” It would seem that the more appropriate term is “signs” as Helmholtz also refers to “Localzeichen” which is uniformly translated as “local signs.” The term *tokens* tends to be used more in English because the *Treatise* is more widely read than his *Popular Lectures*.

Helmholtz emphasised that stereoscopic depth perception is learned and that the invention of the stereoscope “made the difficulties and imperfections of the Innate Theory of sight much more obvious than before” (Helmholtz, 1873, p. 274). Thus, the stereoscope helped to give Helmholtz precisely what he needed to strengthen and defend his own empirical theory of space perception against attacks on it by Hering (see Lenoir, 1993; Turner, 1994). He maintained that not only is binocular depth perception learned but that all spatial perception is founded on judgmental acts based in experience. As Helmholtz saw it, space perception is, from a more general perspective, essentially similar to object recognition.

After 1867, Helmholtz did continue to give popular lectures on vision and, although he did not acquire any further evidence for his empiricism, his opposition to nativism was undiminished. Nonetheless, Boring (1942) dedicated his history of sensation and perception to the long-dead Helmholtz with the prefatory defence that “If it be objected that books should not be dedicated to the dead, the answer is that Helmholtz is not dead” (p. xi).

Helmholtz lived in an era when science was believed to illuminate the path to truth and he did much to chart its course. He searched for principles that would unify the sciences, and these were considered to have their basis in the senses. He early recognised “that knowledge of natural processes was the magical key which places ascendency over Nature in the hands of its possessor” (Helmholtz, 1898, p. 272). There was little room for doubt in this approach, and the uncertainties of modern physics lay ahead. Ironically, it was one of his own students, Max Planck, who laid the foundations for this transformation. Physiology received a radical revision in the late 20th century with the technical innovations that resulted in single-cell recordings, and it has provided support for both Hering’s nativism and Helmholtz’s empiricism (see Turner, 1994). The final section of the *Treatise*, reviewing theories of vision, posed Helmholtz the greatest problems and required the most protracted preparation (see Koenigsberger, 1906, pp. 231–232). Perhaps this is because it led him into the metaphysical domain he had assiduously avoided in his physical and physiological endeavours: “Philosophy, it is true, has been for nearly three thousand years the battle-ground for the most violent differences of opinion, and it is not to be expected that these can be settled in the course of a single life” (Helmholtz, 1898, p. 286). It should be noted that Helmholtz made an explicit distinction between metaphysics and philosophy, and his opinion of the former was not high. He likened their relationship to that between astrology and astronomy concluding: “philosophy, if it gives up metaphysics, still possesses a wide and important field, the knowledge of mental and spiritual processes and their laws” (Helmholtz, 1898, p. 233).

## Popular Lectures

Helmholtz devoted much of his early scientific life to understanding the eye and vision. He not only wrote extensively on the subject but also presented new developments in the field of vision in his *Popular Lectures*. These covered a wide array of topics from the conservation of force to the origin of the planetary system. They were assembled and translated into English in two books (Helmholtz, 1873, 1881); thereafter, several editions of the lectures were published. Later editions of the second series contain an autobiographical sketch by Helmholtz which was originally delivered at the meeting celebrating his 70th birthday in 1891. Two sets of lectures were about vision, one on its recent advances in the first series and the other on its relation to painting in the second series. The three lectures on “The recent progress of the theory of vision” were delivered in 1868 at Frankfurt-am-Main and Heidelberg (see Pastore, 1973; Wade & Finger, 2001), and they followed the structure of his *Treatise*. Helmholtz’s stated aim was:

To render what follows understood in all its bearings, I shall first describe the *physical* characters of the eye as an optical instrument; next the *physiological* processes of excitation and conduction in the parts of the nervous system which belong to it; and lastly, I shall take up the *psychological* question, how mental apprehensions are produced by the changes which take place in the optic nerve. (Helmholtz, 1873, p. 199)

The second series of lectures “On the relation of optics to painting” were delivered in Berlin, Düsseldorf, and Köln between 1871 and 1873. They considered, in turn, form, shade, colour, and harmony of colour. The section on form deals with the paradox of painting—creating the appearance of depth on a flat surface that can be clearly perceived as such. He described the perspectival perception for a single eye but then considered binocular viewing:

We however see the world with two eyes, which occupy somewhat different positions in space, and which therefore show two different perspective views of objects before us. This difference of the images of the two eyes forms one of the most important means of estimating the distance of objects from our eye, and of estimating depth, and this is what is wanting to the painter, or even turns against him; since in binocular vision the picture distinctly forces itself on our perception as a plane surface. (Helmholtz, 1881, p. 80)

Helmholtz points clearly to the conflict between the flatness of the canvas as seen with two eyes and the apparent perspectival depth within it. Moreover, he draws a geometrical parallel between disparities with two eyes and those of motion parallax due to lateral movements of one eye alone:

For it must be observed that as we use different pictures seen with the two eyes for the perception of depth, in like manner as the body moves from one place to another, the pictures seen by the same eye serve for the same purpose. (Helmholtz, 1881, p. 81)

In his lectures on painting, Helmholtz explicitly avoided aesthetic considerations:

We have not here to do with a discussion of the ultimate objects and aims of art, but only with an examination of the action of the elementary means with which it works. The knowledge of the latter must, however, form an indispensable basis for the solution of the deeper questions, if we are to understand the problems which the artist has to solve, and the mode in which he attempts to attain his object. (Helmholtz, 1881, p. 76)

## Conclusion

**Figure 11.**
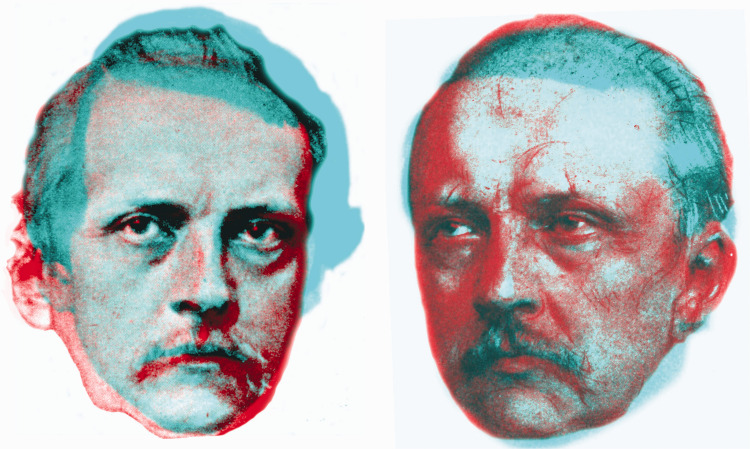
*The faces of Helmholtz* by Nicholas Wade. Two anaglyphic double portraits of Helmholtz from 1848 and 1876 on the left and from 1857 and 1894 on the right.

In his autobiographical sketch, Helmholtz referred to science and art as “the only remaining bond of peace between civilised nations” (1898, p. 267). He also made mention of his personal appearance and representations of it by engravers and sculptors. There are many portraits of Helmholtz so that it is possible to compare them from different stages of his life (Figure 11). While Helmholtz’s physiognomy changed little throughout his adult life, the subjects he examined, such as physiological optics, were transformed by his analyses of them.

**Figure 12.**
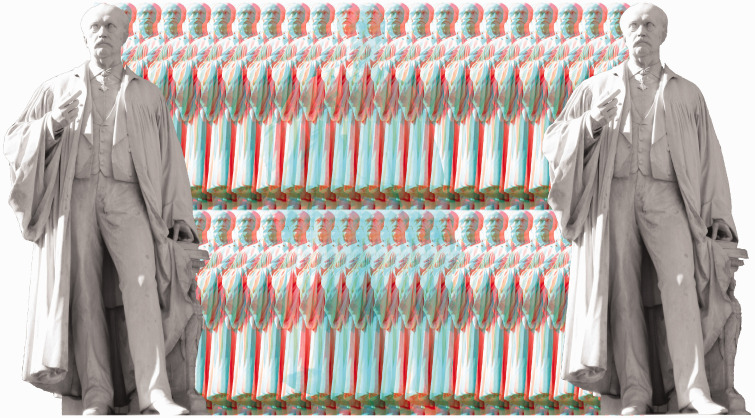
*Helmholtz in depth* by Nicholas Wade. Helmholtz’s statue at the Humboldt University, Berlin, in front of an anaglyphic design composed of multiple copies of it.

Helmholtz received many honours during his life as well as after his death. He was ennobled by the Emperor to von Helmholtz in 1883 and honoured by many universities as well as national scientific societies. Institutes and instruments now bear his name. Figure 12 shows his statue (by the sculptor Ernst Herter) that stands at the entrance to the Humboldt University in Berlin. This final graphical tribute to Helmholtz is itself stereoscopic.
